# Epidemiology of Cutaneous Leishmaniasis Outbreak, Waziristan, Pakistan 

**DOI:** 10.3201/eid2401.170358

**Published:** 2018-01

**Authors:** Mubashir Hussain, Shahzad Munir, Taj Ali Khan, Abdullah Khan, Sultan Ayaz, Muhammad Ameen Jamal, Irfan Ahmed, Sohail Aziz, Noha Watany, Mohamed Kasbari

**Affiliations:** Kohat University of Science and Technology, Kohat, Pakistan (M. Hussain, S. Munir, T.A. Khan, A. Khan);; Abdul Wali Khan University, Mardan, Pakistan (S. Ayaz);; Yunnan Agricultural University, Kunming, China (S. Munir, M.A. Jamal, I. Ahmed);; Khyber Medical University, Peshawar, Pakistan (S. Aziz);; US Naval Medical Research Unit 3, Cairo, Egypt (N. Watany);; French Agency for Health and Safety, Maisons-Alfort, France (M. Kasbari)

**Keywords:** Epidemiology, cutaneous leishmaniasis, leishmaniasis, Leishmania tropica, outbreak, Waziristan, Pakistan, parasites

## Abstract

During 2013–2015, prevalence of cutaneous leishmaniasis in war-affected Waziristan areas was 3.61% by PCR. Youths (1–15 years of age) were more susceptible. Internal transcribed spacer 1 PCR followed by restriction fragment length polymorphism analysis identified *Leishmania tropica* in 215 samples and *Leishmania major* in 6 samples.

Cutaneous leishmaniasis (CL), the most widespread form of leishmaniasis, caused by *Leishmania tropica* and *L. major* (*1*,*2*), has emerged as an endemic disease in Khyber Pakhtunkhwa, Pakistan (*2*–*5*), owing to frequent movement of internally displaced persons (IDPs) from Waziristan in response to surgical strikes and military operations against terrorists by Pakistani armed forces. Keeping in view the impact of the frequent outbreaks of CL in settled areas, we studied the prevalence of CL in war-affected North and South Waziristan, with the help of health department and local government authorities.

We surveyed 7,548 persons from the different endemic areas and collected samples from ulcerating skin lesions from 538 suspected CL patients, 244 in North Waziristan and 294 in South Waziristan, during April 2013–January 2015. To sort out the reservoir, we captured 72 rodents from different locations in Waziristan, such as around houses, in cornfields, and in wild plantations surrounding the houses of CL patients, and analyzed liver and spleen samples by PCR. In addition, we collected sand flies from within 1.5 km of CL patients’ houses for molecular analysis (*6*). We determined the prevalence rate of CL by a formula described previously (*7*) and performed statistical analyses using statistical software SAS Enterprise Guide (version 4.2; SAS Institute, Cary, NC, USA) by univariate analysis of variance with statistical significance at p<0.05.

Prevalence according to sex was consistent with previous findings, indicating that CL infections were more prone to develop in males **(**Table**)** because of more social activity and interaction with IDPs, whereas females always remain covered because of Islamic rules and thus are less prone to sand fly bites. Agewise, we observed a higher prevalence rate in children 0–15 years of age (1.61%) compared with other age groups. Sand flies and rodents were also collected from different endemic villages; none of the trapped rodent samples tested positive for leishmaniasis, but samples from sand flies from 2 endemic villages of North Waziristan (Razmak and Shewa) and 1 endemic village of South Waziristan (Sreykhoray) tested positive by kinetoplast DNA PCR. Samples collected from domestic animals (sheep, goat, cattle, donkey, dogs, mules) were negative for leishmaniasis.

**Table:** Areawise prevalence of cutaneous leishmaniasis in Waziristan, by microscopy and PCR

Area	No. tested	CL-positive samples, no.*			CL prevalence, %	
Microscopy	PCR	Microscopy	PCR
North Waziristan						
Shewa	966	29	35		3†	3.62†
Spinwam	530	17	21		3.2†	3.96†
Mirali	320	6	7		1.87	2.18
Edaky	463	9	11		1.94	2.37
Darpakheil	450	11	14		2.44	3.11†
Hasankheil	373	9	11		2.41	2.94
Dosali	512	19	24		3.71†	4.68†
Miranshah	455	15	17		3.29†	3.73†
Razmak	367	14	16		3.81†	4.35†
Subtotal	4,436	129	156		2.91	3.51†
South Waziristan						
Wanna	412	14	28		3.39	6.79†
Shekai	463	12	18		2.59	3.88†
Jandola	516	10	10		1.93	1.93
Sra Roha	253	8	11		3.16†	4.34†
Makeen	169	4	9		2.36	5.32†
Janata	448	6	11		1.33	2.45
Sreykhoray	195	6	12		3.07†	6.15†
Kotkai	755	12	18		1.58	2.38
Subtotal	3,112	72	117		2.3	3.75†
Total	7,548	201	273		2.66	3.61
*CL, cutaneous leishmaniasis. †Denotes statistically significant difference (p<0.05) analyzed by χ^2^ test. In South Waziristan, the highest prevalence rate (6.79% by PCR), was found in Wanna district; in North Waziristan, Dosali had the highest prevalence (4.68% by PCR). However, South Waziristan showed a higher prevalence rate, 3.75% (117/3,112), than North Waziristan’s 3.51% (156/4,436).						

For this report, we performed internal transcribed spacer 1 PCR followed by restriction fragment length polymorphism analysis for identification of different species of *Leishmania*. For North Waziristan, we observed 63.0% of *L. tropica* and 8% of *L. major* specific bands by this analysis. Similarly, restriction fragment length polymorphism analysis of South Waziristan showed 54% *L. tropica* and 4% *L. major* specific bands. No *L. infantum*–positive cases were found in any human or animal (dog) samples. Moreover, different species of *Phlebotomus* and *Sergentomya* sand fly genera were identified in both North and South Waziristan; *P. sergenti* was the most abundant species, followed by *P. papatasi*. We reported leishmaniasis infection in 6 female *P. sergenti* sand flies*. P. papatasi* is also susceptible to carry *L. tropica* and is widely distributed in different parts of Pakistan, including Khyber Pakhtunkhwa Province (*8*). A previous team had reported *L. infantum* in 2 army personnel deployed in Waziristan, (*9*) but the present detailed study ruled out its presence.

We conclude that CL is prevalent in Waziristan and new cases are increasing day by day. The present study also confirms that *L. tropica* is the causative agent of CL in Waziristan. This study also confirmed that anthroponotic CL caused by *L. tropica* is the main causative agent of CL in Waziristan. All the patients whose specimens tested positive for *L. major* had a history of traveling to zoonotic CL endemic areas of Mezar Sharif (Afghanistan) and Sindh and Balochistan (Pakistan) (*10*). Because of limited access in the study area for security reasons, sampling could not be performed in other endemic parts of Waziristan, so further molecular epidemiologic studies on animal reservoirs and sand flies should be conducted in wider areas of Waziristan, including neighboring tribal and settled areas, to map the complete distribution of the disease.

A leishmaniasis control committee should be established by health authorities in association with the Ministry of Health. It is strongly recommended that proper recordkeeping and documentation systems for leishmaniasis be initiated by health authorities at the local, provincial, and national levels and be well maintained to identify leishmaniasis outbreaks so that control measures can be started well in time. Further, IDP camps must be monitored regularly to minimize the risk that nonendemic areas will be exposed to the disease by infected IDPs.
